# Editorial: The Pulmonary and extra-pulmonary effects of mechanical ventilation in critically-ill patients

**DOI:** 10.3389/fmed.2023.1272322

**Published:** 2023-08-18

**Authors:** Simone Gattarello, Mattia Busana, Gustavo A. Cortes-Puentes

**Affiliations:** ^1^Department of Anesthesia and Intensive Care Medicine, IRCCS San Raffaele Scientific Institute, Milan, Italy; ^2^Department of Anesthesiology, University Medical Center Göttingen, Göttingen, Germany; ^3^Division of Pulmonary and Critical Care Medicine, Mayo Clinic, Rochester, MN, United States

**Keywords:** mechanical ventilation, ventilation induced lung injury (VILI), clinical trial, observational study, positive pressure mechanical ventilation

After more than 70 years since the introduction of positive pressure ventilation into clinical practice, astonishing advances have been made. However, as a frequently observed phenomenon in science and research, the more knowledge one achieves on a specific subject, the more one realizes the enormous distance that we still need to travel to fully understand this process. In fact, despite the significant number of publications focused on the interactions between the lungs, the heart, the kidneys, and the brain during mechanical ventilation, there is still a high degree of uncertainty on how to best approach specific clinical dilemmas related to safety and efficiency of positive pressure ventilatory support.

Among the scientific community, multicenter randomized clinical trials are the gold-standard approach to answer specific medical questions. Undoubtably, the use of randomization and a multicentric approach to research have significantly reduced the risk of bias while conducting any study. Additionally, strictly adhering to the scientific method mitigates unconscious (and often unintended) observations and results that to some extent could selectively favor the study hypotheses initially theorized by the researchers.

Unfortunately, this approach is also associated with specific limitations. The most relevant, in our opinion, is the trade-off toward simplification in the study-hypothesis when designing a clinical trial. Consequently, very often, the study lacks any physiological substrate, which is a crucial driving factor in advancing science and medicine. Comparing “intervention A” with “intervention B” allows us to observe how outcomes vary in a specific study-population, however it does not allow us to understand why. Moreover, despite the efforts of the investigators, the studied population is hardly fully representative, leading to the misconception that what has been observed on a population level will directly apply to each patient we encounter in the clinical practice. Finally, carrying out such big sample size studies is extremely expensive, so the risk that a restricted elite of countries and institutions are the only stakeholders involved in this field of research is very likely.

Observational studies, both prospective and retrospective, are less expensive and easier to organize and manage. Despite being exposed to greater bias due to the absence of randomization and a series of other significant limitations, a greater number of scholars worldwide may have the opportunity to test and divulgate their scientific hypothesis.

A further possibility is offered by the “physiological” study, in which the researcher tries to describe step-by-step the way in which a medical phenomenon manifests itself, both in animals, healthy volunteers or patients. The hardest parts of this approach are (1) the compromise between the invasiveness that the exploration of a clinical phenomenon requires, and (2) the ethics of performing such a study in the critically ill population. Therefore, these studies are often carried out in animals or healthy volunteers, rather than hospitalized individuals, thus reducing the clinical impact of their findings. Even, case-reports can be a worthy example of helpful approach to research: an unusual clinical observation and its clinical management can be the booster for doctors and researchers to hypothesize new studies, or to inform their clinical practice when facing unusual clinical scenarios.

Ideally, we should approach a specific clinical conundrum from multiple viewpoints, with different types of studies and approaches, which allow us to understand the final effects of a specific clinical intervention (clinical trial), the physiological background (physiological studies), and to extrapolate results to patient populations with different characteristics (observational studies). Obviously, the scientific rigor must always be the driver, regardless of the chosen research modality.

This is the approach we took when selecting the contributions for this Research Topic: “*The pulmonary and extra-pulmonary effects of mechanical ventilation*”. Chaudhuri et al. studied the introduction in their department of Non-Invasive Ventilation (NIV) performed with the helmet interface during the COVID-19 pandemic. They found that the helmet was well tolerated and may represent a useful alternative to the more conventional mask interface. Wei et al., studied the use of an electronic microphone to detect a potential selective bronchial intubation. On a murine model, Sparrow et al. found that in prone positioning the interleuchin-6 concentration in some key neural areas is different compared to the supine decubitus. The authors state that this might influence the development and incidence of delirium in the two positions. López-Brull et al. reports in three patients the use of an innovative home device for the delivery of NIV that allows a connection with effort belts. They remind us once more the importance of detecting asynchronies during NIV, both inside and outside the hospital. Finally, we selected for the Research Topic two reviews: the first one (Al-Husinat et al.) focuses on the effects of aspirin, beta blockers, statins and heparin on septic patients during mechanical ventilation. Two second one, from Sood et al. offers us a wide overview on the possible complications in a specific group of mechanically ventilated patients: children.

We hope that reading such articles will generate interest and curiosity among the readers, and, hopefully, these will act as hypothesis generator of new ideas and studies with different designs. Our goal, ultimately, which each single paper, is to try to advance current scientific knowledge, even by a millimeter, or two.

## Author contributions

SG: Conceptualization, Formal analysis, Writing—original draft. MB: Conceptualization, Formal analysis, Writing—original draft. GC-P: Conceptualization, Formal analysis, Writing—original draft.

